# Increased accuracy of genomic predictions for growth under chronic thermal stress in rainbow trout by prioritizing variants from GWAS using imputed sequence data

**DOI:** 10.1111/eva.13240

**Published:** 2021-05-18

**Authors:** Grazyella M. Yoshida, José M. Yáñez

**Affiliations:** ^1^ Facultad de Ciencias Veterinarias y Pecuarias Universidad de Chile Santiago Chile; ^2^ Núcleo Milenio INVASAL Concepción Chile

**Keywords:** accuracy, genomic predictions, GWAS, heat stress, rainbow trout, whole‐genome sequence

## Abstract

Through imputation of genotypes, genome‐wide association study (GWAS) and genomic prediction (GP) using whole‐genome sequencing (WGS) data are cost‐efficient and feasible in aquaculture breeding schemes. The objective was to dissect the genetic architecture of growth traits under chronic heat stress in rainbow trout (*Oncorhynchus mykiss*) and to assess the accuracy of GP based on imputed WGS and different preselected single nucleotide polymorphism (SNP) arrays. A total of 192 and 764 fish challenged to a heat stress experiment for 62 days were genotyped using a customized 1 K and 26 K SNP panels, respectively, and then, genotype imputation was performed from a low‐density chip to WGS using 102 parents (36 males and 66 females) as the reference population. Imputed WGS data were used to perform GWAS and test GP accuracy under different preselected SNP scenarios. Heritability was estimated for body weight (BW), body length (BL) and average daily gain (ADG). Estimates using imputed WGS data ranged from 0.33 ± 0.05 to 0.55 ± 0.05 for growth traits under chronic heat stress. GWAS revealed that the top five cumulatively SNPs explained a maximum of 0.94%, 0.86% and 0.51% of genetic variance for BW, BL and ADG, respectively. Some important functional candidate genes associated with growth‐related traits were found among the most important SNPs, including signal transducer and activator of transcription 5B and 3 (*STAT5B* and *STAT3*, respectively) and cytokine‐inducible SH2‐containing protein (*CISH*). WGS data resulted in a slight increase in prediction accuracy compared with pedigree‐based method, whereas preselected SNPs based on the top GWAS hits improved prediction accuracies, with values ranging from 1.2 to 13.3%. Our results support the evidence of the polygenic nature of growth traits when measured under heat stress. The accuracies of GP can be improved using preselected variants from GWAS, and the use of WGS marginally increases prediction accuracy.

## 1 INTRODUCTION

Climate change, including rising sea levels, changes in water temperatures and increasing frequency and severity of extreme events, will affect fisheries and aquaculture in different ways (Shelton, 2014). The increase in water temperature is one of the most concerning climate change‐related threats for global cold‐water aquaculture species (Callaway et al., 2012; Shelton, 2014). It is expected that aquaculture will face significant challenges, which may affect the stability and sustainability of this business (Callaway et al., 2012). Aquaculture breeding programmes have been extensively focused on the enhancement of growth rate in different species that directly contribute to increased production, but the current fish broodstocks used for aquaculture purposes are adapted to the prevailing environmental conditions and may be suboptimal under future conditions (Sae‐Lim et al., 2017).

Rainbow trout have a narrow optimal temperature that ranges from 12°C to 18°C, with 25°C the upper limit for suitable trout rearing (Raleigh, 1984). Adaptability to increased temperature levels is a result of natural (Chen & Narum, 2020; Chen et al., 2015) and artificial selection (Ineno et al., 2005). In previous studies, performance under increased thermal stress was evaluated in different rainbow trout populations. The presence of additive genetic variation for heat tolerance and growth under heat stress, with heritability values ranging from 0.24 to 0.41 and temperatures from 10°C to 25.7°C (Gallardo‐Hidalgo et al., 2020; Janhunen et al., 2016; Perry et al., 2005), indicates that it is possible to genetically improve these traits in rainbow trout. However, if growth under thermal stress is included in the breeding goal, the phenotypes can only be measured by means of sib testing, and not directly on the selection candidates, similar to disease resistance and carcass quality traits. Thus, genomic information is key for the identification of the genetic architecture and loci involved in the effect of increased thermal stress on growth rate. Genomic tools already available for rainbow trout can be used to better understand the molecular basis and incorporation of genomic information for selective breeding of more robust rainbow trout to future environmental changes (Lhorente et al., 2019).

Genome‐wide association studies (GWASs) are commonly used to dissect the genetic architecture of disease resistance (Correa et al., 2016; Geng et al., 2015; Rodríguez et al., 2019; Tsai et al., 2016; Yáñez et al., 2019) and growth‐related traits (Gonzalez‐Pena et al., 2016; Gutierrez et al., 2015; Reis Neto et al., 2019; Tsai et al., 2015; Wringe et al., 2010; Yoshida et al., 2017) in different aquaculture species. The polygenic genetic architecture of some economic traits was observed, with no major single nucleotide polymorphism (SNP) surpassing the genome‐wide significance threshold and many markers explaining a small proportion of the genetic variance. The use of whole‐genome sequence (WGS) data is expected to improve the detection of quantitative trait loci (QTLs), because such data should contain most causal mutations, providing a much higher resolution (Sanchez et al., 2017; Van Den Berg et al., 2019). However, it is expensive to sequence a large number of animals; therefore, genotype imputation from low density to WGS would be a cost‐effective approach. Using WGS as a reference population, studies have reported an imputation accuracy higher than 0.80 for cattle (Bouwman & Veerkamp, 2014; Fernandes Júnior et al., 2021) and pigs (Van Den Berg et al., 2019).

Information from thousands of markers can be incorporated into genetic evaluations to estimate genomic breeding values (GEBVs) through genomic prediction (GP) schemes, including traits controlled by a high number of quantitative trait loci (QTLs) with small effects, such as growth‐related traits. GP strategies have revolutionized most breeding schemes globally, including aquaculture species, particularly for traits that are expensive or impossible to measure in selection candidates (Sonesson et al., 2009). Previous studies have compared the use of GP with the conventional pedigree‐based best linear unbiased prediction (PBLUP) for different aquaculture species, and found an increase in relative accuracy for GP, independent of the trait, SNP density or statistical method used (Bangera et al., 2017; Barría et al., 2018; Correa et al., 2017; Tsai et al., 2016; Yoshida, Bangera et al., 2018, Yoshida, Carvalheiro et al., 2018; Yoshida, Carvalheiro et al., 2019, Yoshida, Lhorente et al., 2019). To further increase the accuracy of GP, recent studies have suggested the use of WGS data, due to the potential incorporation of causal mutations (Ni et al., 2017; Wiggans et al., 2017). However, it has been shown that marginal or no increase in prediction accuracy is obtained when comparing the use of WGS to high‐density (HD) SNP panels in *Drosophila melanogaster*, cattle, chicken and simulation studies (van Binsbergen et al., 2015; Brøndum et al., 2015; Hayes et al., 2014; Heidaritabar et al., 2016; Ni et al., 2017; Ober et al., 2012; Pérez‐Enciso et al., 2015). In contrast, the prioritization of SNPs, by preselection them from WGS data, could be an option to significantly increase GP prediction accuracy (Moghaddar et al., 2019; Raymond et al., 2018; van den Berg et al., 2016).

The objectives of this study were to (i) perform a genome‐wide association study to dissect the genetic architecture, and identify molecular markers and candidate genes associated with growth traits in rainbow trout; and (ii) investigate the accuracy of genomic predictions based on imputed WGS, using different scenarios of preselected variants from GWAS for growth traits under chronic heat stress in rainbow trout.

## 2 MATERIALS AND METHODS

### 2.1 Upper‐thermal challenge test and phenotypes

In this study, we used rainbow trout from the 2016 year class of the breeding nucleus owned by Effigen S.A. A detailed description of the origin and management for the population is presented in previous studies (Barria et al., 2019; Yoshida, Carvalheiro et al., 2018, 2019). The upper‐thermal challenge was performed at Aquainnovo's Aquaculture Technology Center Patagonia, Puerto Montt, Chile. A total of 1829 animals from 119 families (average of 15 fish/family and a range from 12 to 16 fish/family) were acclimated in a single tank with fresh water for 17 days (~14°C), and then, fish were equally distributed into three tanks of 7 m^3^, with a similar representation of each family per tank. The temperature was increased gradually (1°C/day) until the 9th day and then kept between 18 and 22°C for 62 days. Water flow was maintained at a rate of 1 to 1.5 L per hour and the oxygen saturation in the water was kept above 80% during the experiment. The fish received two weekly treatments of seawater at 10 ppt to avoid opportunistic bacterial infections and the challenge test spanned 62 days, with mortality recorded daily. More details about the breeding programme and the upper‐thermal challenge were described by Yoshida, Bangera et al. (2018) and Gallardo‐Hidalgo et al. (2020), respectively.

We used body length (BL, in cm) and body weight (BW, in g) after the challenge as growth traits for further genetic evaluation, and the initial body length (IBL, in cm) and initial body weight (IBW, in g) at the beginning of the challenge as covariates, respectively. Additionally, we also used the average daily gain (ADG, in g) = (BW − IBW)/(final age − initial age) as the growth trait measured during the challenge test.

### 2.2 Genotypes and imputation to whole‐genome sequences

Genomic DNA was extracted and purified from fin clip samples of 956 fish using the DNeasy Blood & Tissue Kit (Qiagen) according to the manufacturer's protocol. A total of 764 and 192 samples were genotyped with a customized 1 K and 26 K SNP panels (SeqSNP), respectively. The SNPs were selected from a denser SNP panel (Gao et al., 2018; Palti et al., 2015) to be evenly distributed across the Omyk_1.0 reference genome for *Oncorhynchus mykiss* (GenBank Assembly Accession GCA_013265735.3 USDA_OmykA_1.1). The samples were genotyped using the targeted genotyping by sequencing SeqSNP technology (Zhang et al., 2020) developed by LGC (Biosearch Technologies Genomic Analysis by LGC). Samples genotyped with both 1 K and 26 K SNP panels were sequenced using the NextSeq 500 system and 75‐bp single‐read run mode, resulting in ~137 million reads (average of ~180.5 K reads per sample) and ~660 million reads (~3.4 million reads per sample), and averaged an effective target SNPs covered of 161x and 102x for 1 K and 26 K, respectively. The final VCF file was filtered separately for the samples genotyped with the 1 K and 26 K SNP panels using Plink v1.90 (Purcell et al., 2007), and the exclusion criteria are as follows: Hardy–Weinberg equilibrium (HWE, *p*‐value 10^−6^), minor allele frequency (MAF) <0.01, and genotyping call rate for SNPs and samples of <0.70. A total of 0.37 K and ~10 K SNPs from 613 and 192 animals were retained, respectively.

Genomic DNA was extracted from fin clips of 102 parents (36 males and 66 females) from the challenged population using the DNeasy Blood & Tissue Kit (Qiagen) according to the manufacturer's instructions and submitted to the Beijing Genomics Institute (BGI, China) for whole‐genome sequencing using DNBseq technology. Raw sequencing data were aligned to the *O*. *mykiss* genome (GenBank Assembly Accession GCA_013265735.3 USDA_OmykA_1.1) consisting of 2.18 GB of total sequence comprising 1228 contigs with a contig N50 of 15.5 Mb. The Burrows–Wheeler Aligner (BWA) analysis tools (Li & Durbin, 2010) were used to map the reads of each sample to the reference genome resulting in a mapping rate of samples ranging from 97.51% to 98.16%, and effective mapping depth between 10.31x and 17.65x. For SNP calling, the standard protocol implemented in the Genome Analysis Toolkit (GATK, https://www.broadinstitute.org/gatk/) was used. The final VCF file consisted of a set of 22.6 million nonredundant variants from 102 rainbow trout. Quality control was performed with Plink v1.90 (Purcell et al., 2007) using the following thresholds: HWE (*p*‐value 10^−8^), MAF <0.01 and call rate for SNPs <0.80, retaining a total of ~3.2 million SNPs from 102 samples.

To assess the overall imputation accuracy (*r* ^2^ = squared correlation between true and imputed genotypes) and remove variants with low imputation accuracy, we randomly divided the resequenced animals into five cross‐validation sets: five exclusive reference sets with 80% of animals genotyped with ~3.2 million SNPs and validation sets with 20% of animals genotyped with 10 K SNPs masking the remaining SNPs from WGS genotypes. After imputation, a total of 1,821,336 SNPs with *r* ^2^ value greater than 0.80 were retained as the reference data set for the final imputation.

Stepwise genotype imputation was used to perform the final imputation from 613 animals genotyped with 0.37 K SNPs to 10 K SNPs using 192 animals as the reference data set (step 1), and 805 individuals (613 + 192 animals) with 10 K SNPs were imputed to ~1.8 million SNPs using 102 animals as the reference (step 2). FImpute v. 3.0 (Sargolzaei et al., 2014) was used to perform all genotype imputations. A postimputation quality control (HWE <*p*‐value 10^−8^ and MAF <0.05) resulted in a total of 1,390,748 imputed SNPs available for 850 individuals denoted as imputed WGS genotypes.

Rainbow trout sampling procedures were approved by the Comité de Bioética Animal from the Facultad de Ciencias Veterinarias y Pecuarias, Universidad de Chile (Certificate No. 19270‐VET‐UCH).

### 2.3 Genome‐wide association analysis

The GWAS was performed using the weighted single‐step genomic best linear unbiased prediction (wssGBLUP) method (Wang et al., 2012) implemented in BLUPF90 family programmes (Misztal et al., 2018). The following model was used:

(1) y=Xβ+Za+e

where y is a vector of phenotypes (ADG, BL or BW); *β* is a vector of tank as fixed effect for all traits and body length and body weight at initial challenge test for BL and BW as covariate, respectively; ***a*** is a vector of additive genetic effects that follows a normal distribution $∼N(0,\;H\sigma_{a}^{2})$, where $\sigma_{a}^{2}$ is the additive variance and **H** is the kinship matrix (Aguilar et al., 2010); X and Z are incidence matrices for ***β*** and ***a*** effects, respectively; and e is the vector of random error with a distribution $∼N(0,\;I\sigma_{e}^{2})$, where I is the identity matrix and $\sigma_{e}^{2}$ is the residual variance. In the wssGBLUP, the pedigree relationship matrix **A^−1^** is replaced by matrix **H^−1^** (Aguilar et al., 2010), which combines genotype and pedigree relationship coefficients:

(2) $$H^{‐1}\;{\text{= A}}^{‐1}\text{+}\left[\begin{array}{cc} 0 & 0\\ 0 & G^{‐1}‐A_{22}^{‐1} \end{array}\right]$$

where $A_{22}^{‐1}$ is the inverse of a pedigree‐based relationship matrix for genotyped animals and $G^{‐1}$ is the inverse genomic relationship matrix. In the wssGBLUP, the marker variances were estimated from allele frequencies and used as weights, which were updated at each iteration (Wang et al., 2014). In the first iteration, all weights assumed an initial value of one, which corresponds to the single‐step genomic BLUP (ssGBLUP, Figure S1) method (Misztal et al., 2009). As suggested by Wang et al. (2012) and Zhang et al. (2016), we used the second iteration of wssGBLUP to get final SNP effect estimates. For GWAS analysis, we included all animals imputed to WGS, all the phenotyped fish present in Table 1, and pedigree information from 115,647 animals.

**TABLE 1:** Descriptive statistics for growth‐related traits under chronic upper‐thermal stress in rainbow trout

Traits	*N*	Mean	Min	Max	SD	CV (%)
Genotyped animals						
Age (days)	805	544	529.00	553.00	7.89	1.45
ADG (g)	805	3.40	−0.11	7.84	1.26	22.76
BL (cm)	804	29.58	18.00	41.30	2.24	7.56
BW (g)	805	409.42	165.00	645.00	93.17	36.98
Phenotyped animals						
Age (days)	1024	536	473.00	563.00	17.04	3.18
ADG (g)	1024	3.17	−1.51	9.80	1.33	25.58
BL (cm)	1024	28.61	19.00	41.30	2.50	8.74
BW (g)	1024	370.44	136.00	636.00	94.74	41.96
ALL						
Age (days)	1829	539	473.00	563.00	14.34	2.66
ADG (g)	1829	3.27	−1.51	9.80	1.30	24.77
BL (cm)	1828	29.04	18.00	41.30	2.44	8.39
BW (g)	1829	387.60	136.00	645.00	96.00	39.84

Abbreviations: ADG, average daily gain; BL, body length; BW, body weight.

The top five SNPs that explain the largest proportion of genetic variance for each trait were selected as the lead SNP and used to search for candidate genes based on a window within 100 kb upstream and downstream of each lead SNPs to be considered putative candidate genes associated with the trait. The gene search was performed using BLAST (Basic Local Alignment Search Tool) against the *Oncorhynchus mykiss* reference genome (Omyk_1.0, GenBank Assembly Accession GCA_002163495.1).

### 2.4 Genetic parameters and heritability

The total additive genetic variance ($\sigma_{a}^{2}$) and residual variance ($\sigma_{e}^{2}$) were estimated using the kinship matrices **A** and **H** for pedigree‐based BLUP (PBLUP) and ssGBLUP using imputed WGS, respectively. The following equation was used to compute the heritability for each growth‐related trait:

(3) $$h^{2}=\frac{\sigma_{a}^{2}}{\sigma_{a}^{2}+\sigma_{e}^{2}}$$

### 2.5 Genomic prediction

Genomic prediction was evaluated using four different scenarios with different SNP densities and strategies for marker selection. The objective was to test the best scenario in terms of accuracy of prediction. In the WGS scenario (WGS), all available markers from imputed WGS genotypes were used. The 50 K pruned scenario (50K_pruned) was selected to produce a subset of markers proportionally distributed across the genome according to chromosome size, as evenly spaced as possible and in similar levels of linkage disequilibrium (LD) with each other SNP (Cleveland & Hickey, 2014). Thus, the SNPs were selected based on the option ‐‐indep‐pairwise of Plink v1.90 software (Purcell et al., 2007) using the following parameters: a window size of 40 kb, a step of one SNPs and a variable linkage disequilibrium according to the chromosome. Additionally, the GWAS results were used to select the most important 50 K and 1 K SNPs based on the descending order of the estimated genetic variance explained by each SNP from wssGBLUP analysis per trait (scenario 50K_wssGBLUP and 1K_wsGBLUP). To reduce the potential biases in accuracy of genomic prediction in these scenarios, the GWAS analysis to preselected SNPs was based on five repetitions of a fivefold cross‐validation scheme to estimate SNP effects.

We used the BLUPF90 family of programmes (Misztal et al., 2018) to perform the genetic evaluations using pedigree‐based information (PBLUP) and the genomic relationship matrix BLUP (GBLUP) (VanRaden, 2008). The statistical models fitted were the same as Equation 1, except for the vector **a** of random additive genetic polygenic effects that follows a normal distribution $∼N(0,\;A\sigma_{a}^{2})$ or $∼N(0,\;G\sigma_{a}^{2})$, for, respectively, pedigree relationship matrix **A** in PBLUP and genomic relationship matrix G in GBLUP, as described by VanRaden (2008).

The predictive abilities of pedigree‐ and genomic‐based models were assessed using the same subgrouping of the fivefold cross‐validation used to select the most important SNPs in scenarios 50K_wssGBLUP and 1K_wsGBLUP. All genotyped and phenotyped animals (*n* = 805) were randomly divided into five exclusive data sets, determined one at a time, where 80% of the animals were used as the training data set to estimate the SNP effects, and the phenotypes of the remaining 20% of the animals were masked and used as the validation set.

To evaluate the performance of each scenario and model, the accuracies were measured in the validation sets using the following equation (Ødegård et al., 2014):

(4) $$r_{\text{GE}\text{BV,}\text{BV}}\text{=}\frac{r_{\text{GEBV,y}}}{h}$$

where $r_{\text{GEBV,y}}$ is the correlation between the EBV or GEBV of a given model (predicted for the validation set using information from the training set) and the phenotype, while h is the square root of heritability.

## 3 RESULTS

### 3.1 Phenotypes, genotypes and genetic parameters

A total of 1829 fish were phenotyped, and 805 were genotyped. The average age was 539 days at the end of the challenge for all phenotyped fish, the ADG was 3.3 g (SD = 1.3 g), BL was 29 cm (SD = 2.4 cm) and BW was 388 g (SD = 96 g) (Table 1).

For WGS genotype data, the call‐rate parameter excluded the highest number of SNPs (~16.8 million), and for 26 K and 1 K SNP panels ~3.3 K and 94 SNPs were discarded, respectively (Table 2). The HWE filtered the lower number of markers: 40 from the 1 K SNP panel and ~638.6 K from WGS. All fish samples passed call‐rate quality control for WGS data and 26 K SNP panel. For the 1 K SNP panel, 613 samples remained for final analysis.

**TABLE 2:** Summary results from genotype quality control of whole‐genome sequence (WGS) data, imputed WGS data, and 26 K and 1 K single nucleotide polymorphism (SNP) panels for rainbow trout

Parameters	Genotype data sets			
	WGS^a^	Imputed WGS^b^	26K^c^	1K^c^
Initial samples	102	102	192	764
Initial SNPs	22,649,022	1,821,336	26,000	1000
Minor allele frequency	2,045,912	245,564	12,520	496
Call rate	16,771,535	–	3358	94
Hardy–Weinberg equilibrium	638,649	185,024	364	40
Final samples	102	102	192	613
Final SNPs	3,192,926	1,390,748	9758	370

^a^ Minor allele frequency (MAF) <0.01, call rate <0.80 and Hardy–Weinberg equilibrium (HWE) <1e^−8^.

^b^ MAF <0.05 and HWE <1e^−8^.

^c^ MAF <0.01, call rate <0.70 and HWE <1e^−6^.

A uniform MAF distribution and a right‐skewed distribution were observed for imputed WGS genotypes (MAF average = 0.23) and the 50K_pruned scenario (MAF average = 0.21), respectively (Figure S2). In contrast, for both 50K_wssGBLUP and 1K_wssGBLUP, for all growth traits, the MAF histograms showed a left‐skewed distribution (Figures [Link], [Link], [Link]). The distribution of 50K_wssGBLUP MAF is similar among the traits, considering the average of five repetitions of a fivefold cross‐validation scheme more than 75% of SNPs presented MAF value higher than 0.27, and a mean value of 0.34, whereas the MAF distribution of SNPs in 1K_wssGBLUP scenarios differed from 50K_wssGBLUP due to the absence of SNPs with MAF lower than 0.12, a larger number of SNPs with MAF higher than 0.31, and mean MAF higher than 0.37.

The additive genetic variance and heritability were slightly higher when using the pedigree‐based method compared with the imputed WGS. For instance, heritability values using PBLUP and ssGBLUP, respectively, were 0.40 and 0.36 for ADG, 0.39 and 0.33 for BL, and 0.59 and 0.55 for BW, with a reduction in the standard error of heritability for ssGBLUP compared with PBLUP (Table 3).

**TABLE 3:** Estimates of variance components and heritability values for growth traits in rainbow trout estimated by pedigree‐based BLUP (PBLUP) and single‐step GBLUP (ssGBLUP)

Traits	$\sigma_{a}^{2}$	$\sigma_{e}^{2}$	*h* ^2^	$\sigma_{a}^{2}$	$\sigma_{e}^{2}$	*h* ^2^
	PBLUP			ssGBLUP		
ADG	0.703	1.053	0.400 (0.065)	0.623	1.131	0.355 (0.053)
BL	1.738	2.753	0.387 (0.065)	1.447	2.975	0.327 (0.054)
BW	5935.10	4049.80	0.594 (0.074)	5525.20	4557.10	0.548 (0.055)

Abbreviations: ADG, average daily gain; BL, body length; BW, body weight.

$\sigma_{a}^{2}$: additive genetic variance; $\sigma_{e}^{2}$: residual variance; *h* ^2^: heritability (standard error).

### 3.2 Genome‐wide association analysis

**FIGURE 1:**
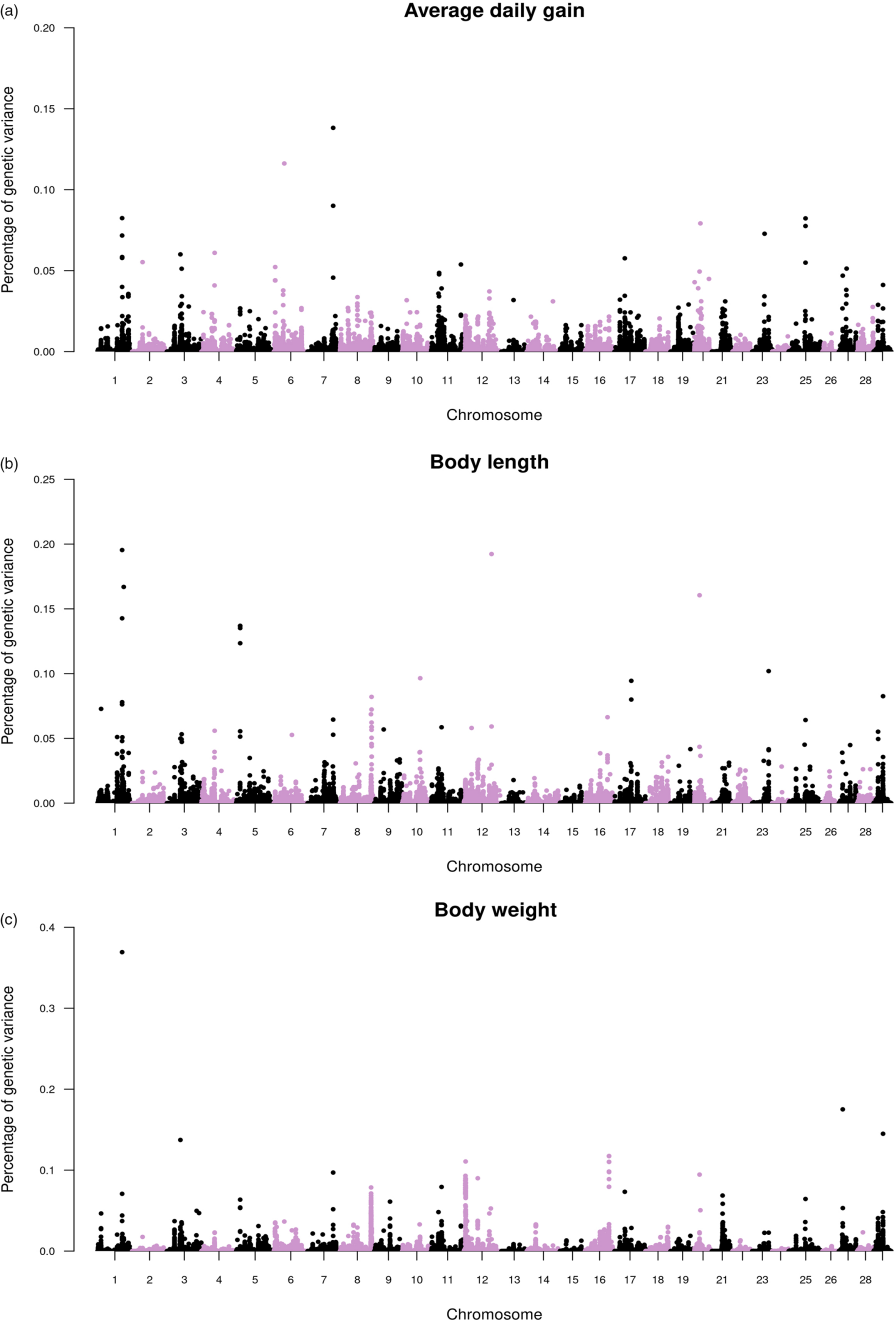
Manhattan plot of percentage of genetic variance explained by each SNP using the wssGBLUP approach for (a) average daily gain, (b) body length and (c) body weight under chronic upper‐thermal stress in rainbow trout

Manhattan plots for the proportion of genetic variance explained by each SNP estimated using imputed WGS data for growth‐related traits under chronic thermal stress are shown in Figure 1. After the second iteration of wssGBLUP, the top five SNPs explained a total genetic variance ranging from 0.51 to 0.95% (Table 4).

**TABLE 4:** Top five ranked SNPs explaining the largest proportion of genetic variance and the closest candidate genes associated with growth‐related traits under chronic upper‐thermal stress based on wssGBLUP in rainbow trout

Chr^a^	Position	Pvar^b^	Candidate genes^c^
Average daily gain			
07	62669267	0.1382	RHOA, CISH, FBXO42, SLC25A34, TMEM82, SPEN, GDI1, TWF2, RPUSD1, QRICH1, TMCC1, LOC110528409, LOC110528419, LOC110527144
06	25326130	0.1162	LOC110525677^d^, UBE2D4, TCF7L1B, TGOLN2, ZGC:153044
07	62654174	0.0901	PRKAR2A^d^, CISH, RHOA, FBXO42, SLC25A34, TMEM82, SPEN, GDI1, TWF2, RPUSD1, QRICH1, TMCC1, LOC110528409, LOC110527144, LOC110528419
01	59530229	0.0824	DNTT^d^
25	40665614	0.0823	–
Body length			
01	59514997	0.1954	DNTT^d^
12	67283722	0.1923	STAT5B^d^, STAT3, PLCL2, LOC110538193, LOC110538955
01	63435956	0.1669	PYGB, ABHD12, APMAP, ACSS1, VSX1, ENTPD6, BANF1
20	12073241	0.1605	FRA10AC1^d^, RBP4, MYOF, PLCE1, SLC35G1, LGI1, CEP55L, NUDT13, PDE6C
01	59513201	0.1427	DNTT^d^, BLNK
Body weight			
01	59513201	0.3693	DNTT^d^, BLNK
27	9075818	0.1751	IRF2BP2^e^, POLR1G^d^, PRX, PLD3, FOXA2, NOVA1, GPR4, HIPK4, LOC110507470, LOC110507475
29	22572225	0.1449	SMYD1, FABP1, PRRC2B, EDF1, LZTS3, FASTKD5, DQX1, SPR, LOC110509833, LOC110509836, LOC110509837
03	33016645	0.1373	–
16	58446630	0.1174	SLC17A9^d^, TASOR, ASB14B, DNAH12, RPS23, GID8, BIRC7, YTHDF1, LOC110492512

^a^ Chromosome.

^b^ Percentage of genetic variance.

^c^ Based on Omyk_1.0 as reference genome for *Oncorhynchus mykiss*.

^d^ Gene intercepted by SNP on intronic region.

^e^ Gene intercepted by SNP on exonic region.

Some genes were found to be intercepted by the top five SNPs for each growth traits, located in exonic or intronic regions (Table 4), as cAMP‐dependent protein kinase type II‐alpha regulatory subunit (*PRKAR2A*) for ADG: signal transducer and activator of transcription 5B (*STAT5B*) for BL, and RNA polymerase I subunit G (*POLR1G*), interferon regulatory factor 2‐binding protein 2 (*IRF2BP2*) and solute carrier family 17 member 9 (*SLC17A9*) for BW. A top SNP for ADG and BW, and two top SNPs for BL on Omy01 intercept the gene DNA nucleotidylexotransferase (*DNTT* or *TDT*) on intronic regions. Also, some biologically relevant genes located within 100 kb upstream and downstream of each top five SNP are available in Table 4. These genes are potential candidate genes associated with growth‐related traits under chronic thermal stress, including ras homolog family member A (*RHOA*) and cytokine‐inducible SH2‐containing protein (*CISH*) both located on chromosome Omy07, and associated with ADG. For BL, we identified signal transducer and activator of transcription 3 (*STAT3*) on Omy12, glycogen phosphorylase brain form (*PYGB*) and lysophosphatidylserine lipase *ABHD12* (*ABHD12*) both on Omy01, and retinol‐binding protein 4 (*RBP4*) and myoferlin (*MYOF*) on Omy20. Both the fatty acid‐binding protein (*FABP1*) and histone‐lysine N‐methyltransferase *Smyd1* (*SMYD1*) on Omy29 were associated with BW.

### 3.3 Prediction accuracy

The summary statistics and sum of the proportion of genetic variance captured by each marker were estimated separately for each trait in different scenarios based on wssGBLUP (Table 5). The 50K_pruned scenario resulted in the lowest sum of the proportion of genetic variance captured by the selected SNPs, with sum values ranging from 10.2% to 14.2% for ADG and HW, respectively. In contrast, the selection of the most important 50K_wssGBLUP SNPs captured more than 78% of the proportion of genetic variance, while the 1K_wssGBLUP SNPs captured at least 15.4%, but notably increased the mean of the percentage of genetic variance explained, for example from 0.0016 to 0.0154 in 50K_wssGBLUP and 1K_wssGBLUP scenarios, respectively for ADG.

**TABLE 5:** Summary statistics for the percentage of genetic variance explained by SNPs selected in each genotype scenario for growth‐related traits under chronic upper‐thermal stress in rainbow trout

Genotype scenarios	Traits	Sum^a^	Mean	Min	Max
		Percentage of genetic variance			
50K_pruned	ADG	10.18	0.0002	0.0000	0.1382
	BL	11.60	0.0002	0.0000	0.1954
	BW	14.23	0.0003	0.0000	0.3693
50K_wssGBLUP	ADG	78.26	0.0016	0.0004	0.3806
	BL	80.97	0.0016	0.0003	0.3603
	BW	87.33	0.0017	0.0002	0.4931
1K_wssGBLUP	ADG	15.44	0.0154	0.0078	0.3806
	BL	18.63	0.0186	0.0090	0.3603
	BW	24.70	0.0247	0.0101	0.4931

Abbreviations: ADG, average daily gain; BL, body length; BW, body weight.

^a^ Sum of the estimated genetic variance captured in descending order for each genotype subset.

**FIGURE 2:**
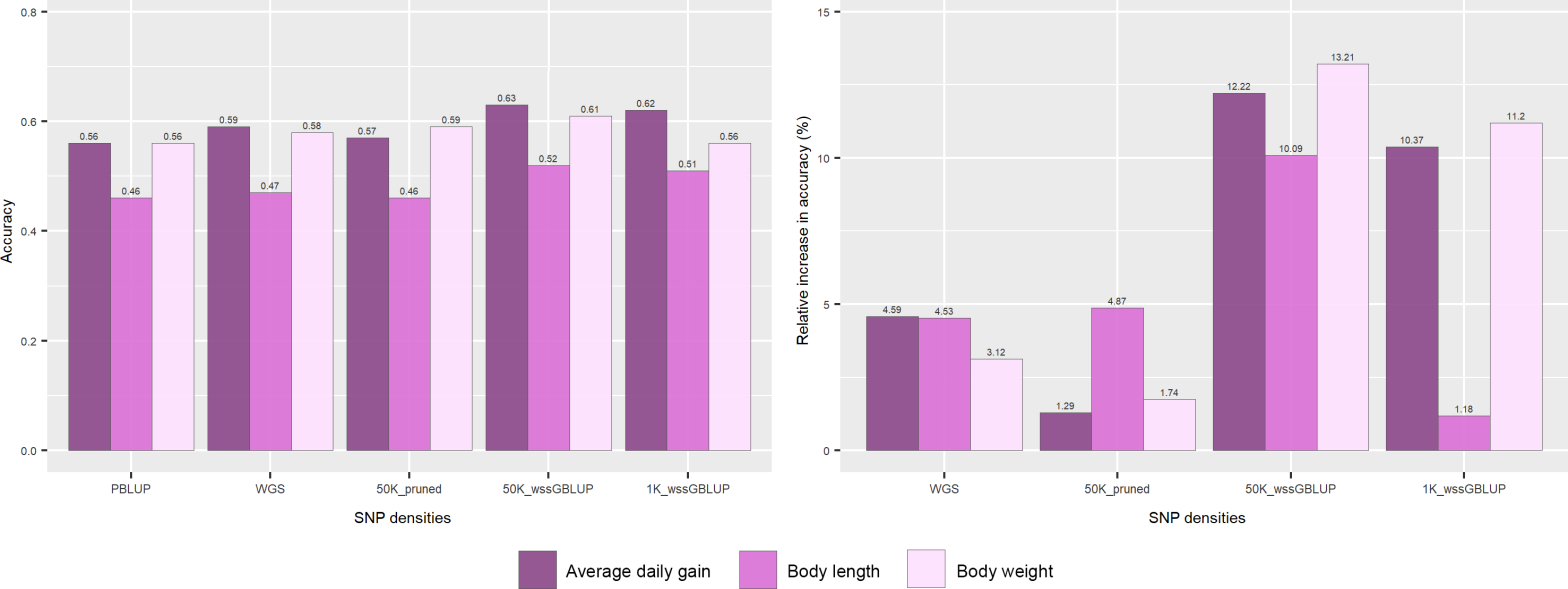
(a) Accuracy of selection using pedigree BLUP (PBLUP), whole‐genome sequence (WGS) and different densities of genotype subsets (50K_pruned, 50K_wssGBLUP and 1K_wssGBLUP). (b) Relative increase in accuracy (%) of genomic selection using imputed WGS and different densities of genotype subsets (50K_pruned, 50K_wssGBLUP and 1K_wssGBLUP) compared with PBLUP for growth traits under chronic upper‐thermal stress in rainbow trout

Based on the five repetitions of fivefold cross‐validation, the prediction accuracy for GEBV from genomic methods outperformed the accuracy for EBV from PBLUP, independent of the genotype scenario (Figure 2a,b). The accuracy of predicted GEBV was slightly higher for WGS and 50K_pruned than for PBLUP, with values ranging from 3.12% to 4.59% and 1.29% to 4.87%, respectively. Compared to PBLUP, the relative increase in accuracy for 50K_wssGBLUP was at least 10.1% for all traits and ranged from 1.2 to 11.2% for 1K_wssGBLUP (Figure 2b). For ADG and BW, the accuracy values were similar for both 50K_wssGBLUP and 1K_wssGBLUP, whereas for BL, the accuracy was considerably higher for 50K_wssGBLUP compared with 1K_wssGBLUP.

## 4 DISCUSSION

### 4.1 Phenotypes and genetic parameters

Salmonids have adapted to cold water and, in the case of rainbow trout, are optimally reared at temperatures up to 20°C (Mäkinen, 1994; Wurtsbaugh & Davis, 1977). A lower feed intake, faster growth and better feed conversion ratio at lower temperature (14°C and 15°C) compared with high temperature (20°C) were reported by Janhunen et al. (2016) and Codabaccus et al. (2013) in rainbow trout. The optimum temperature for growth performance is 17°C with a fast decline in growth rate at temperatures higher than 20°C (Mäkinen, 1994; Wurtsbaugh & Davis, 1977). Furthermore, the water temperature must be considered an important parameter in meat quality. There is higher adipose deposition when fish are reared at high temperatures (>18°C) and fatty acid bioconversion capacity is reduced (Johnston et al., 2000; Mellery et al., 2016).

However, rainbow trout can adapt to increased temperatures as a result of natural (Chen et al., 2015) or artificial selection (Ineno et al., 2005), suggesting the presence of additive genetic variation for thermal tolerance in trout populations. A rainbow trout strain that can feed actively and grow normally at 24°C was established after 14 generations of selection at Miyazaki Prefecture in Japan (Ineno et al., 2005). In addition, a recent study compared growth rate of rainbow trout exposed to an upper‐thermal challenge (between 20°C and 22°C) to fish reared at lower temperatures (approximately 7°C), and a significant difference of ~100 g (*t* test; *p* < 0.05) was found in favor of fish reared at increased temperatures (Gallardo‐Hidalgo et al., 2020). They also reported the heritability of 0.41 (±0.15) for body weight using PBLUP for fish under increased thermal challenge and 0.59 (±0.17) for fish at low water temperature conditions. The genetic correlation was 0.76 (±0.07) between these two traits. The authors suggested that in both temperature conditions there was significant genetic variation with a slight effect of heat stress in the quantitative genetic basis of growth traits in this particular population of rainbow trout. In addition, in some studies where rainbow trout were evaluated under normal water temperature conditions, the estimated heritability values for growth traits using a pedigree‐based method ranged from 0.35 to 0.43 (Janhunen et al., 2016; Leeds et al., 2016), whereas estimated heritability using genomic information was reported to range from 0.30 to 0.62 (Ali et al., 2020; Gonzalez‐Pena et al., 2016; Reis Neto et al., 2019). Our heritability estimates are within the range of values previously reported, suggesting a significant additive genetic variation for growth traits under chronic heat stress in rainbow trout.

### 4.2 GWAS analysis

Growth‐related traits including body weight and average daily gain are among the most important economic traits in selective breeding for aquaculture species. Genetic improvement, by means of artificial selection, can reduce time and cost to produce market‐size fish. However, these traits are complex in nature and are generally controlled by several genes (Goddard & Hayes, 2009). Dissecting the genetic architecture of the target traits is important to define the best genomic methods to apply in commercial breeding programmes (i.e. genomic selection or marker‐assisted selection). Most GWASs have identified the polygenic nature of growth traits in fish, as in Atlantic salmon (Gutierrez et al., 2015; Tsai et al., 2015; Yoshida et al., 2017), rainbow trout (Gonzalez‐Pena et al., 2016; Reis Neto et al., 2019; Wringe et al., 2010), Nile tilapia (Yoshida, Carvalheiro et al., 2019, Yoshida, Lhorente et al., 2019; Yoshida & Yáñez, 2020) and catfish (Li et al., 2018). We increased the number of variants from low density to 1.39 million SNPs through genotype imputation aimed to achieve a better GWAS resolution. The use of imputed SNPs to WGS in cattle was effective at detecting significant peaks not previously found when high‐density chip was used in GWAS analyses (Sanchez et al., 2020; Wang et al., 2020), but in our case, no significant SNP was found when we used the ssGBLUP method (Figure S1). Furthermore, we found numerous markers in almost all chromosomes explaining a small percentage of the genetic variance through wssGBLUP (Figure 1a–c), with no evidence of major QTL for the three growth traits studied here in rainbow trout. Thus, our results support the evidence of the polygenic architecture of these traits.

Based on the top five SNPs that explained the highest proportion of genetic variance, we found several genes that could potentially be involved in ADG, BL and BW. For instance, two SNPs associated with ADG and located in Omy07 were found to be close to the *RHOA* and *CISH* genes. The *RHOA* gene has been shown to mediate transforming growth factor‐β (*TGF*‐β) activity, a potent regulator of cell growth and differentiation in many cell types (Chen et al., 2006). *RHOA* signalling is critical to *TGF*‐β‐induced smooth muscle cell differentiation (Chen et al., 2006) and is important for the regulation of cytoskeletal dynamics in numerous cell types (Tzima, 2006). microRNA 133 (miR133) negatively interacts with *RHOA*, affecting skeletal muscle functions in pearl oyster (*Pinctada martensii*) (Zheng et al., 2016), whereas the upregulation of *CISH* in white muscle was reported in a selectively bred line for fast growth in rainbow trout (Cleveland et al., 2020), suggesting that the expression of *CISH* was induced by immune stimulants and is a negative feedback regulator of growth hormone (*GH*) signalling (Maehr et al., 2014).

The second most important SNP associated with BL was found on Omy12, in an intronic region of *STAT5B* and close to the *STAT3* gene. *STAT5B* is suggested to be an important modulator of *GH*, *GH* receptor, insulin‐like growth factor I (*IGF*‐*I*), prolactin and the insulin signalling pathway, which are involved in growth and reproduction traits (Ji et al., 1999; Kloth et al., 2002; Sadeghi et al., 2012; Woelfle et al., 2003). Mutations in the *STAT5B* gene may result in primary *IGF*‐*I* deficiency and *GH* insensitivity (Rosenfeld et al., 2007). In chicken, two SNPs in *STAT5B* were found to be associated with early body weight and egg weight, suggesting a promising marker for use in marker‐assisted selection in this species (Sadeghi et al., 2012). In Nile tilapia, the overexpression of *STAT5B* can neutralize the effects of *GH* overexpression, suggesting the partial role of *STAT5B* on the deleterious effects of *GH* overexpression observed in genetically selected fish (Marins et al., 2002).

The requirement of *STAT5B* for sexual dimorphism of body growth rate was reported in mice and zebrafish (Huang et al., 2018; Udy et al., 1997; Zhang et al., 2012). The disruption of *STAT5B* affects the expression of a subset of sex‐dependent genes in the liver of male zebrafish, compared with female, reducing the number of male‐ and female‐biased genes in *STAT5B* mutant zebrafish. Significant growth inhibition was observed when targeting the *STAT3* gene using antisense oligonucleotides directed against the translation initiation site (Rubin Grandis et al., 1998). In juvenile *STAT3* mutant zebrafish, a dramatic alteration in the number of genes involved in immune and infection response, skeletal development, somatic cell growth and downregulated expression of the collagen gene family was observed. In addition, *STAT3* mutant zebrafish showed severe lateral and vertical curvature of the spine, spine fracture and incomplete bone joints with a narrower junction between vertebrae at an early juvenile stage (Xiong et al., 2017).

On Omy20, a SNP associated with BL was found close to both *RBP4* and *MYOF*. *RBP4* is involved in the regulation of insulin and insulin resistance, which would affect fetal growth (Chan et al., 2011; Yang et al., 2020). Zebrafish exposed to aromatase inhibitor prochloraz presented significantly downregulated mRNA expression of *RBP4* and changed protein concentrations related to mitochondrial energy metabolism in ova, leading to the subsequent decrease in body length of larval offspring (Dang et al., 2018). In Berkshire pigs, *RBP4* was suggested to be a candidate gene for production traits, due to its significant effect on ADG and back fat thickness (Do et al., 2012). In addition, the presence of two SNPs in *RBP4* might negatively influence the birth weight, BW and ADG in 6‐ and 12‐month‐old Chinese cattle (Wang et al., 2010).

*MYOF* is important for muscle development and regeneration, and might reduce muscle mass and *MYOF* null mice suggesting a defect in muscle growth similar to that produced from manipulation of *IGF*‐*I* or its receptor (Doherty et al., 2005). *MYOF* is a critical mediator of postnatal muscle growth mediated by *IGF*‐*I* in mice, and the loss of functional *MYOF* can retard muscle growth, by a *IGF*‐*I* nonresponse (Demonbreun et al., 2010). In grass carp (*Ctenopharyngodon idellus*), *MYOF*, along with other genes, may play an important role in muscle hardening (Larsson et al., 2012; Xu et al., 2020; Yu et al., 2014).

We found a SNP associated with BW on Omy27 intercepting an exonic region of the *IRF2BP2* gene. This gene is a skeletal and cardiac muscle‐expressed ischaemia‐inducible activator of vascular endothelial growth factor A (*VEGFA*) that may contribute to revascularization of ischaemic cardiac and skeletal muscles (Teng et al., 2010). *IRF2BP2* controls osteoclast and osteoblast differentiation via Kruppel‐like factor 2 (*KLF2*) in mice (Kim et al., 2019). *SMYD1* found on Omy29 is also associated with BW and plays a role in myogenesis. Deletion of *SMYD1* impaired myoblast differentiation resulting in fewer myofibres and decreased expression of muscle‐specific genes in zebrafish (Nagandla et al., 2016). In *Xenopus laevis*, the expression of *SMYD1* is necessary for muscle cell formation (Kawamura et al., 2008).

Some SNPs were found in intronic regions of *PRKAR2A*, *DNTT*, *FRA10AC1*, *POLR1G* and *SLC17A9* genes (Table 4). The function of these genes has not been clearly reported to be involved with growth‐related traits; therefore, the function of the associated SNPs and genes must be better characterized in salmonids. *PRKAR2A* has been associated with some types of cancer, such as lung adenocarcinoma (Bidkhori et al., 2013), breast, colorectal and various human nonendocrine cancers (Vincent‐Dejean et al., 2008). Three different SNPs on chromosome Omy01 intercepted the *DNTT* gene, that is associated with DNA repair and the random addition of a small number of nucleotides to unpaired DNA regions during V(D)J recombination (Fowler et al., 2013; Sarac & Hollenstein, 2019). In mice, *DNTT* has been suggested to participate in memory and learning processes (Peña De Ortiz et al., 2003). *SLC17A9* is thought to be a disease‐related gene associated with disseminated superficial actinic porokeratosis, a rare autosomal dominant genodermatosis (Cui et al., 2014), gastric carcinoma (Li et al., 2019) and colorectal cancer (Yang et al., 2019).

### 4.3 Genomic prediction

Genomic prediction is currently implemented in several aquaculture breeding programmes for different species. Previous studies reported the benefits of genomic predictions in terms of increased accuracy of selection ranging from 4% to 20% over pedigree‐based selection for growth traits (Palaiokostas et al., 2018; Tsai et al., 2015, 2017; Yoshida, Bangera et al., 2018, Yoshida, Carvalheiro et al., 2018; Yoshida, Carvalheiro et al., 2019, Yoshida, Lhorente et al., 2019). Our results show that the use of genomic information for estimating breeding values increased accuracy from 1.18% to 13.21% compared with only using pedigree information, varying by trait and the genotype scenario (Figure 2). In addition, a slight increase in genomic prediction accuracy was observed when comparing WGS‐imputed genotyped data and the 50K_pruned scenario. In simulation studies, it was suggested that WGS data could improve the accuracy of genomic prediction by up to 31%, depending on heritability, statistical method, MAF and QTL density (Druet et al., 2014; Iheshi ulor et al., 2016). Increased predictive ability is expected due to the inclusion of most of the causal mutations in WGS data, and decreased limitation due to LD between SNPs and causal mutations in the predictions (Meuwissen & Goddard, 2010). However, as in the present study, previous works in cattle (Frischknecht et al., 2018; Hayes et al., 2014; Raymond et al., 2018; van Binsbergen et al., 2015), sheep (Moghaddar et al., 2019) and chicken (Heidaritabar et al., 2016; Ni et al., 2017) suggest none to marginal increase in genomic prediction accuracy when comparing the use of WGS vs. a dense SNP panel. This supports the hypothesis that whole‐genome sequence data for genomic prediction may be unnecessary for genomic prediction.

There are plausible explanations for the results of genomic prediction using WGS data. First, when WGS data are used most of the SNPs are in LD with a large number of noncausal mutations, which do not allow for the capture of genetic variance in genomic regions that control the target trait (Van Den Berg et al., 2017; van Binsbergen et al., 2015). Second, the genotype imputation may generate some false‐positive associations. The redundant information for two or more SNPs in high LD may generate noise (Song et al., 2019) affecting the accuracy of genomic prediction (van Binsbergen et al., 2015). Furthermore, the accuracy of imputation can be low for some chromosomes or regions, due to incorrect anchoring of SNPs or errors in the physical map, which can reduce the detection of causal mutations and affect the accuracy of prediction (Dufflocq et al., 2019; Yoshida, Bangera et al., 2018, Yoshida, Carvalheiro et al., 2018). Or third, the small number of animals in the training set for genomic prediction is not enough to estimate the SNP effects and achieve the highest accuracies. For high densities SNP chips, the accuracy of genomic prediction would keep increasing as the number of records increases, making the use of large data sets to estimate the markers effects necessary to fully take advantage of high‐density SNPs (Meuwissen, 2009).

We also created a 50K_pruned scenario, in which SNPs are in approximated LD with each other, and two other scenarios which incorporated only the most important SNPs selected a priori from WGS imputed data. SNPs were chosen based on the level of genetic variance explained for each of the three traits, better exploiting the potential direct or indirect relationship with causal mutations. Our results demonstrate that 50K_pruned is actually a selection of WGS imputed data with 10% to 14% of genetic variance explained, and much lower average and minimum value of percentage of genetic variance compared with 50K_wssGBLUP. These results suggest that the pruned selection of a 50 K SNP panel leads to the inclusion of SNPs with importance equal or near zero (Table 4), generating noise that ultimately affects the accuracy of genomic prediction. In contrast, genomic prediction accuracies increased when using the 50K_wssGBLUP and 1K_wssGBLUP scenarios, compared with WGS imputed data, 50K_pruned and PBLUP. Similar results were found in real and simulated data for dairy cattle and sheep (Moghaddar et al., 2019; Raymond et al., 2018; van den Berg et al., 2016), reinforcing that the use of SNPs in LD with potential causal loci was more efficient in genomic prediction than the use of all markers together or randomly selected SNPs. Using a similar approach to preselect SNPs from GWAS performed with WGS data, Song et al. (2019), Calus et al. (2016), Veerkamp et al. (2016) and Lu et al. (2020) reported no increase in accuracy of genomic predictions for pig, dairy cattle or Japanese flounder (*Paralichthys olivaceus*), probably due to the genetic architecture of traits, limited ability to correctly estimate QTL or used more independent populations for preselected SNPs and genomic predictions. Furthermore, in some populations with small effective population size and long‐range LD, it might be difficult to increase the accuracy of genomic prediction, as in Holstein cattle (MacLeod et al., 2014) and some species and breeds, which have been under strong selection (Druet et al., 2014).

Variation in accuracy between ADG, BL and BW was expected, especially due to the different heritability values of the traits. For ADG and BW, within‐trait differences observed for the 50K_wssGBLUP and 1K_wssGBLUP scenarios were almost nonexistent. However, within‐trait differences were significantly higher for BL in 50K_wssGBLUP versus 1K_wssGBLUP. Including more genetic variants is expected to increase the fraction of genetic variation explained (Table 4), resulting in higher prediction accuracy until a plateau is reached. For both ADG and BW, a similar change in accuracy was observed when using 1K_wssGBLUP or 50K_wssGBLUP. In the case of BL, filtering the most important 1 K SNPs would not benefit accuracy. These results highlight that the SNP density needed to achieve accuracy plateaus is dependent on the scenario used to select the SNPs and the genetic architecture of the trait. Previous studies for disease resistance in aquaculture species assessed genomic prediction accuracy using low marker density, randomly selected from denser SNP panels (ranging from 27 K to 50 K) (Bangera et al., 2017; Correa et al., 2017; Yoshida, Bangera et al., 2018, Yoshida, Carvalheiro et al., 2018). Results indicate that a 3 K to 20 K SNP array is necessary to achieve similar accuracy values to those obtained when using a denser SNP panel. In the present study, selecting markers that are most likely to be in high LD with causal mutations facilitated the reduction in SNP density while maintaining the accuracy of genomic predictions close to the 50K_wssGBLUP values. Furthermore, VanRaden et al. (2011) suggested that the differences in genomic accuracy between low and high‐density SNPs may be more evident with increased sample size.

Some studies have suggested an inverse relationship between the SNP effect and allele frequencies (Abdollahi‐Arpanahi et al., 2014; Park et al., 2011). Park et al. (2011) reported that in humans about 50% of the genetic variance for height was captured by SNPs with a MAF value higher than 0.10. However, in dairy cattle the proportion of genetic variance captured using 50 K SNPs was approximately 80%, with a high proportion of QTL with moderate frequency affecting milk yield traits (Haile‐Mariam et al., 2013). In our study, the comparison between the mean MAF value for both 50 K scenarios suggested that the proportion of genetic variance is better captured by common variants than for rare variants. Furthermore, Druet et al. (2014) and Van Den Berg et al. (2016) reported that an increase in the prediction accuracy is expected for WGS data if causative mutations are primarily rare variants.

GWAS of rainbow trout using imputed WGS data confirmed the polygenic architecture of growth traits under increased thermal stress. Interestingly, we identified candidate genes related to ADG, BL or BW under increased thermal stress in rainbow trout, providing a better understanding of the molecular basis of growth under heat stress. Accuracy was not greatly improved when using imputed WGS data to perform genomic prediction compared with 50K_pruned. In contrast, a considerable increase in the accuracy of genomic predictions was observed when 50 K and 1 K SNPs were preselected based on GWAS and compared to PBLUP, WGS data and 50K_pruned. Our results may be associated with the same population used to select SNPs and for genomic predictions. Even using a cross‐validation strategy, fish from the training and validation set are probably related. Therefore, further studies using different population sets would be useful to further validate the use of preselected SNPs from GWAS for genomic predictions.

## CONFLICT OF INTEREST

Both authors declare no conflict of interest.

## AUTHOR CONTRIBUTIONS

GMY designed the study, performed the analyses and wrote the manuscript. JMY designed the study and contributed to the analysis. Both authors have reviewed and approved the manuscript.

## Supporting information

Fig S1

Fig S2

Fig S3

Fig S4

Fig S5

## Data Availability

The data that support the findings of this study are openly available in the Figshare Repository at https://doi.org/10.6084/m9.figshare.13482564. The *Oncorhynchus mykiss* reference genome is publicly available at NCBI (Omyk_1.0, GenBank Assembly Accession GCA_002163495.1, https://www.ncbi.nlm.nih.gov/genome/?term=GCA_002163495.1).
