# Supplementary figures and images for: Increased accuracy of genomic predictions for growth under chronic thermal stress in rainbow trout by prioritizing variants from GWAS using imputed sequence data
Source: Evol Appl. 2021 May 18;15(4):537–52. doi: 10.1111/eva.13240 (PMC9046923; doi:10.1111/eva.13240)

(A)

Average daily gain

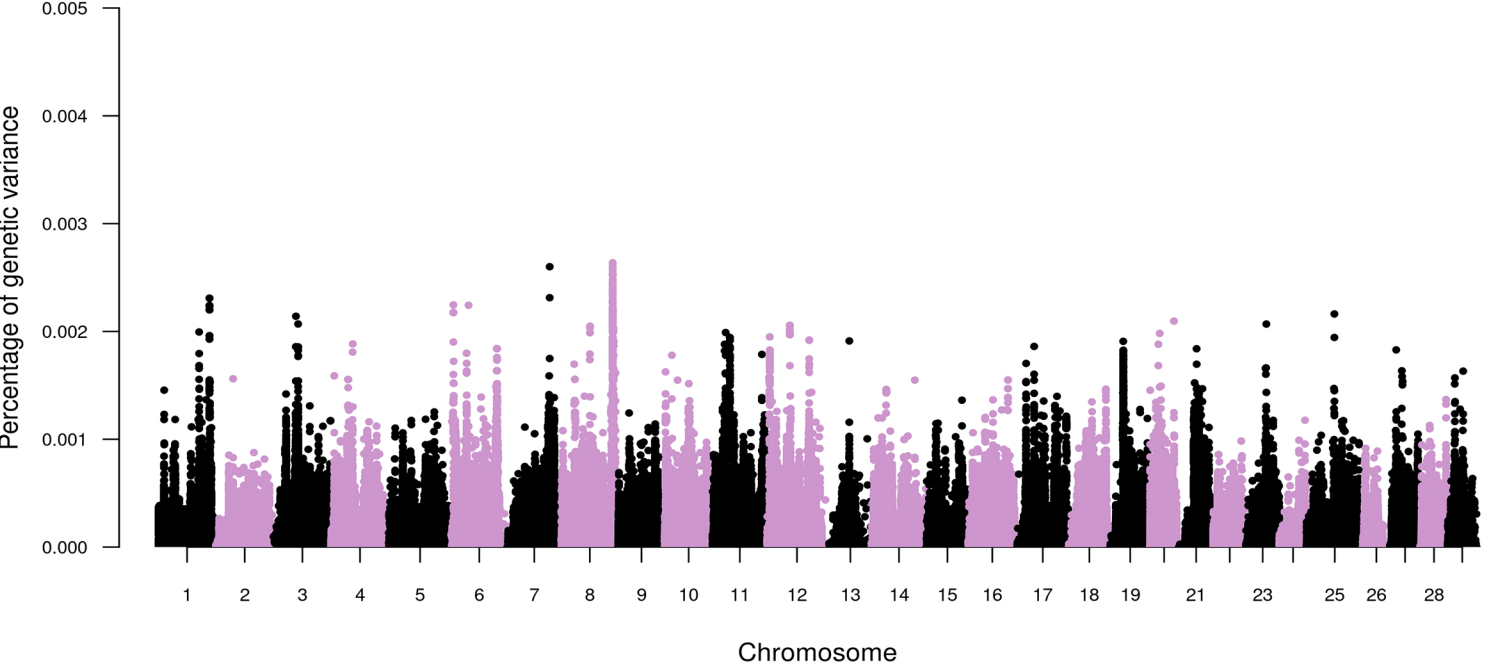

(B)

Body length

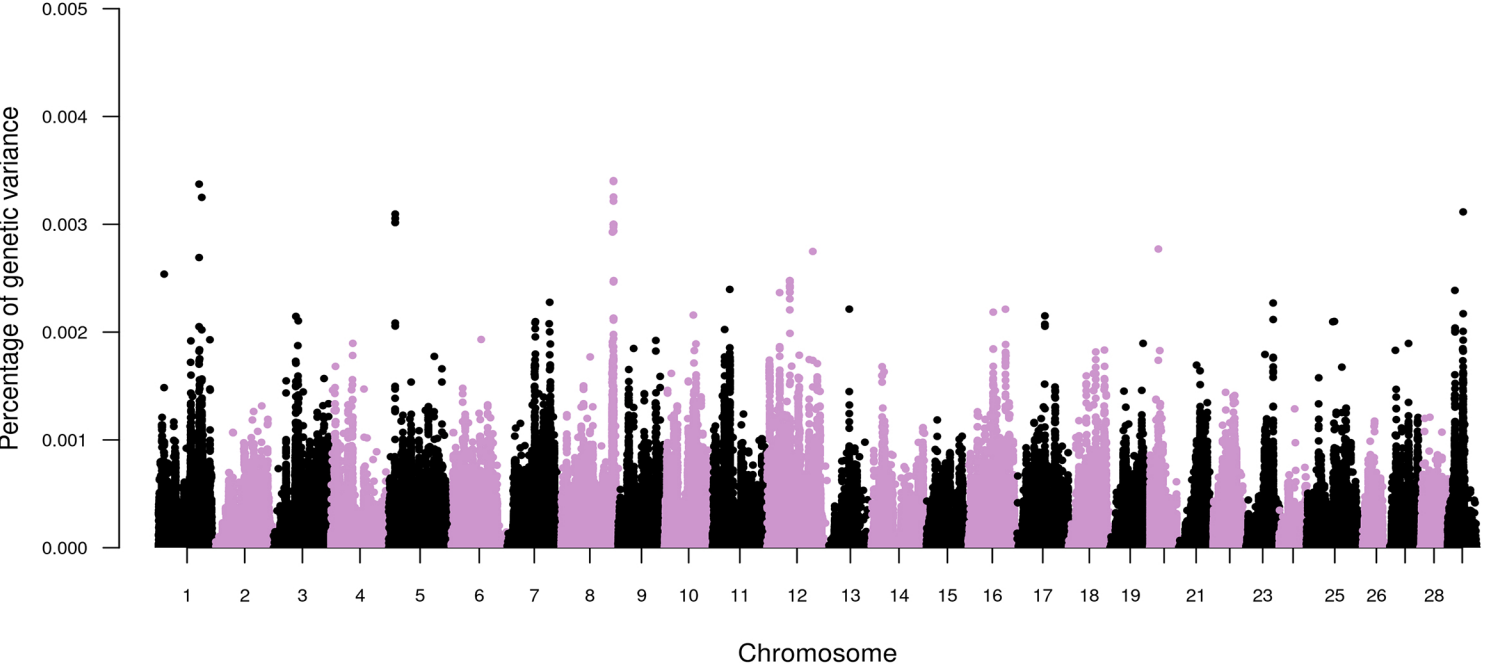

(C)

Body weight

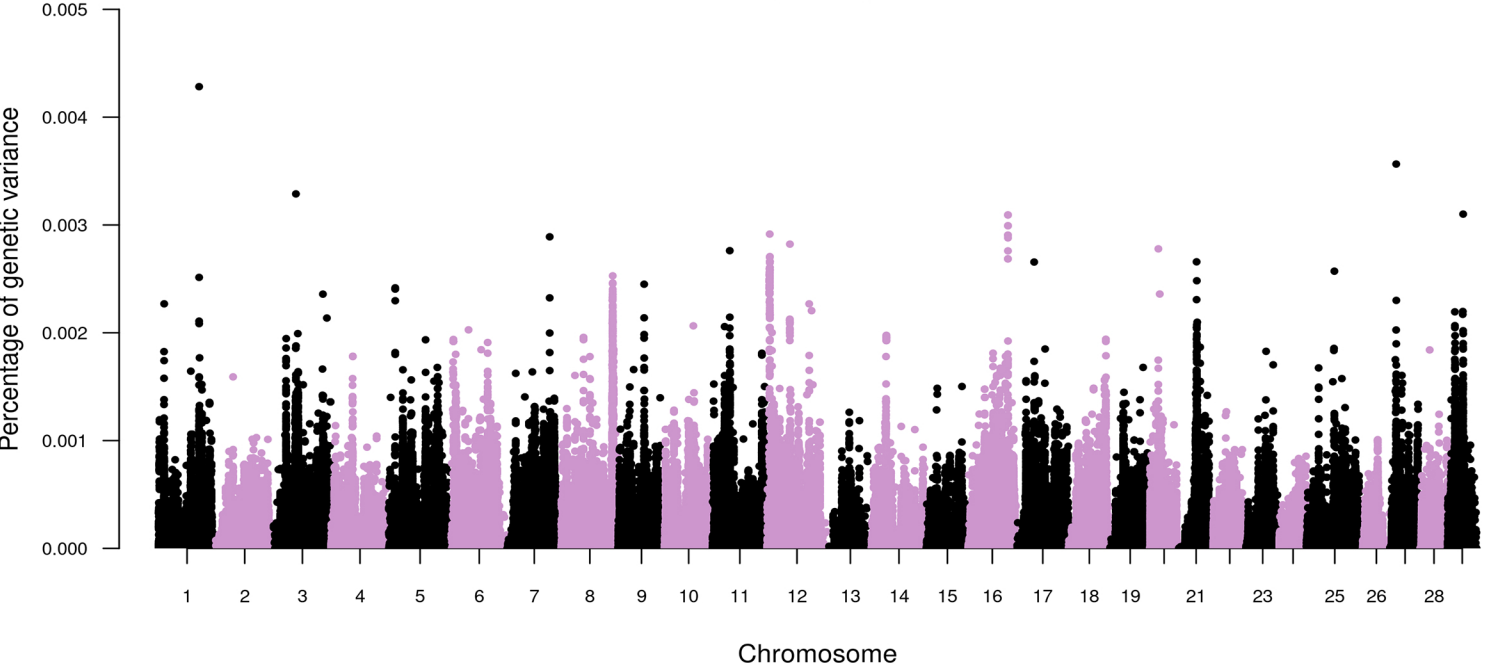

Supplement: Supplementary file 1 — Fig S1 [file EVA-15-537-s002.pdf]

(A) - WGS

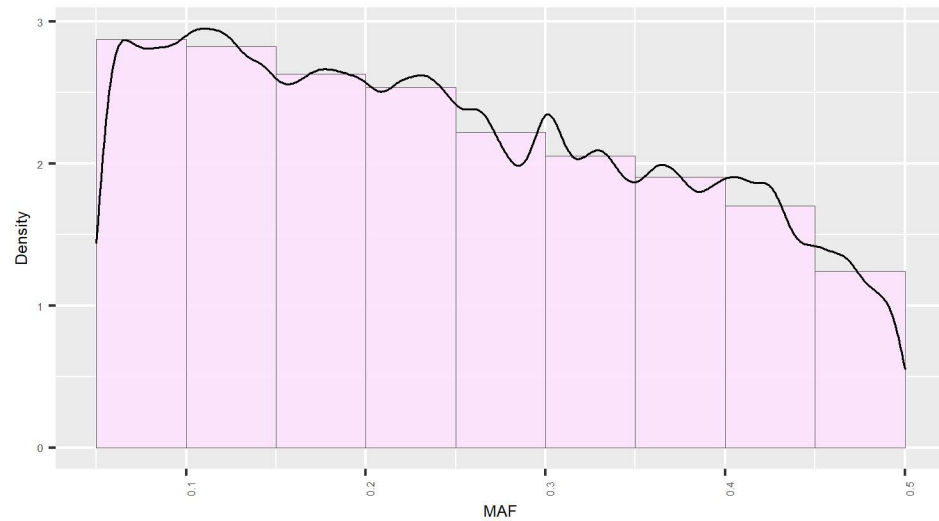

(B) - 50K\_pruned

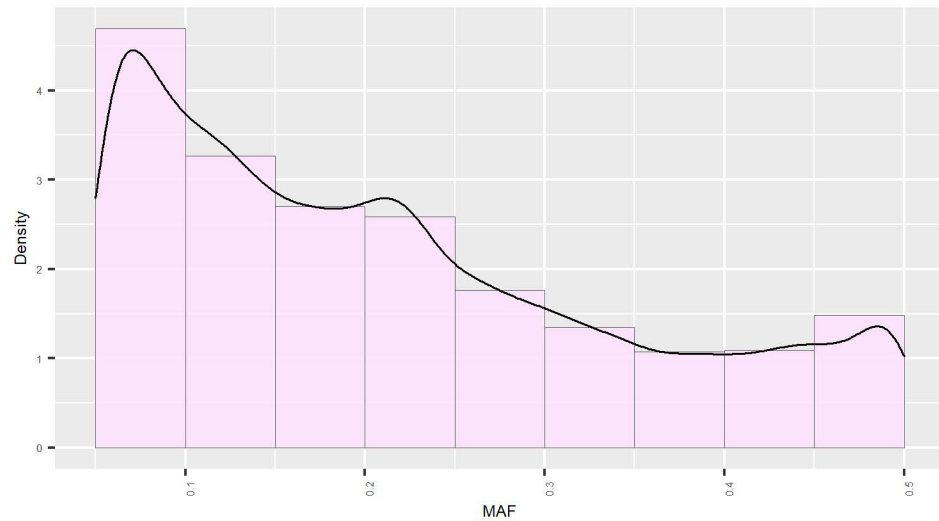

Supplement: Supplementary file 2 — Fig S2 [file EVA-15-537-s001.pdf]

**(A) - Average daily gain - 50K\_wssGBLUP**

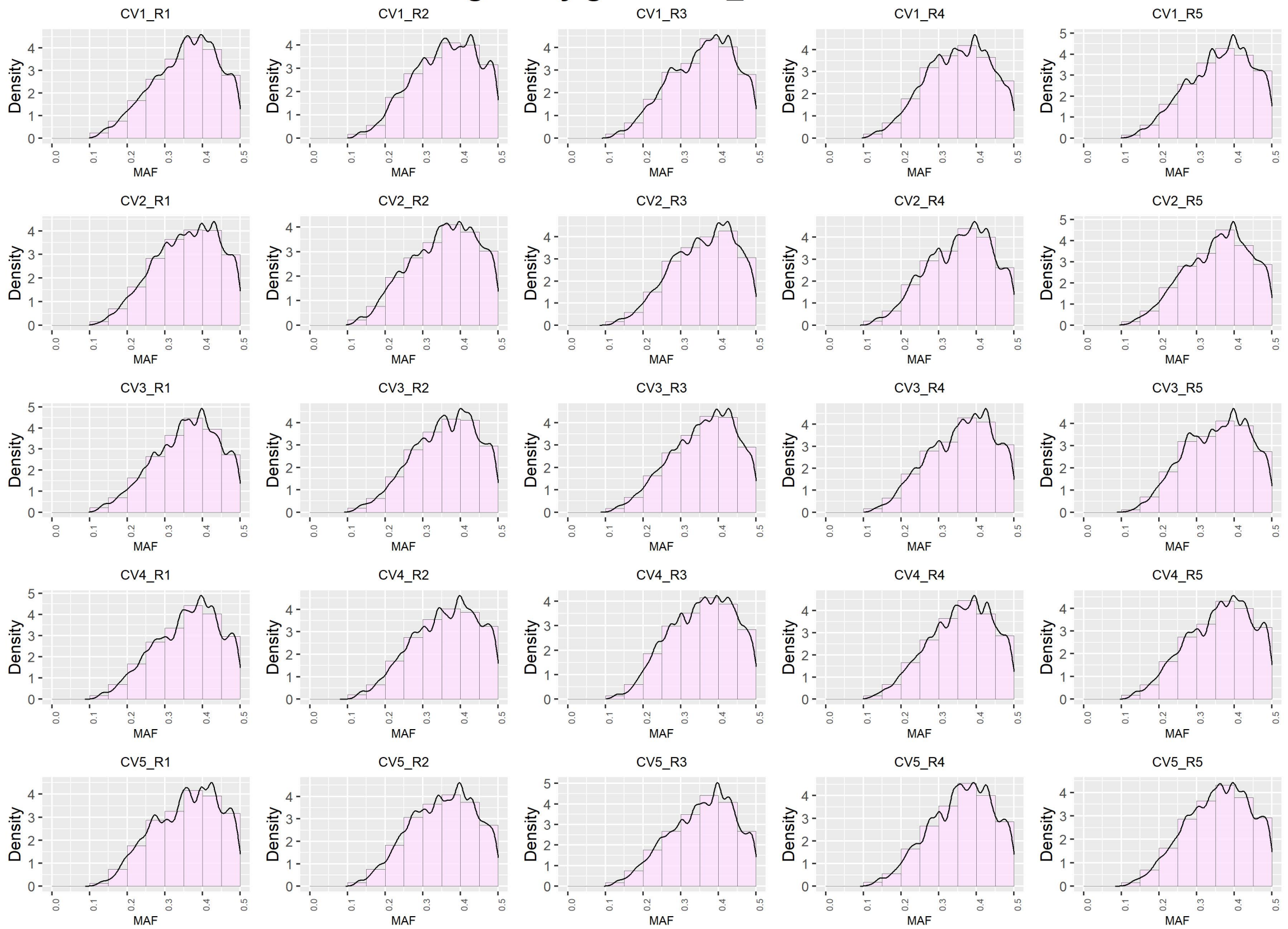

**(B) - Average daily gain - 1K\_wssGBLUP**

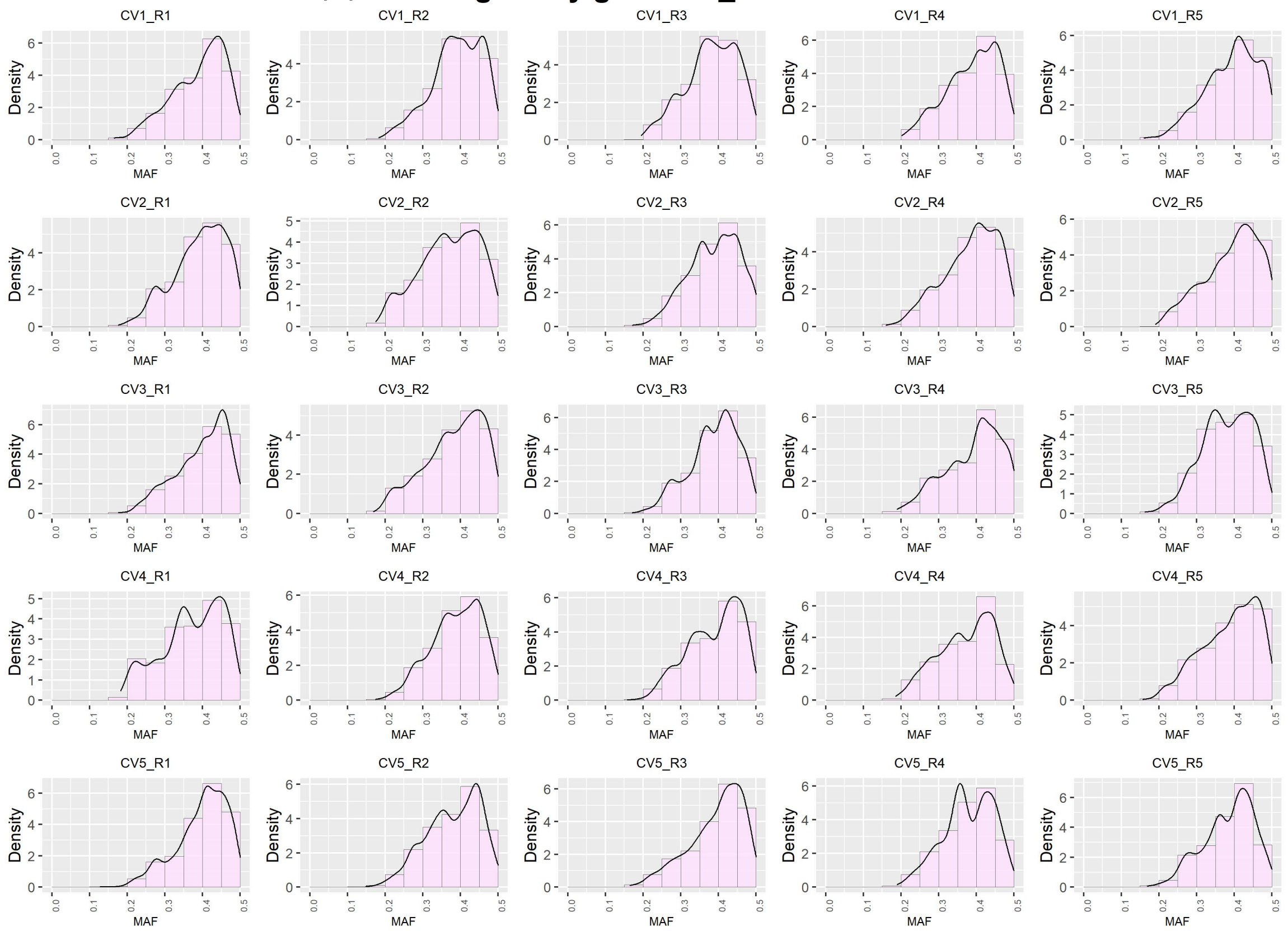

Supplement: Supplementary file 3 — Fig S3 [file EVA-15-537-s005.pdf]

## (A) - Body length - 50K\_wssGBLUP

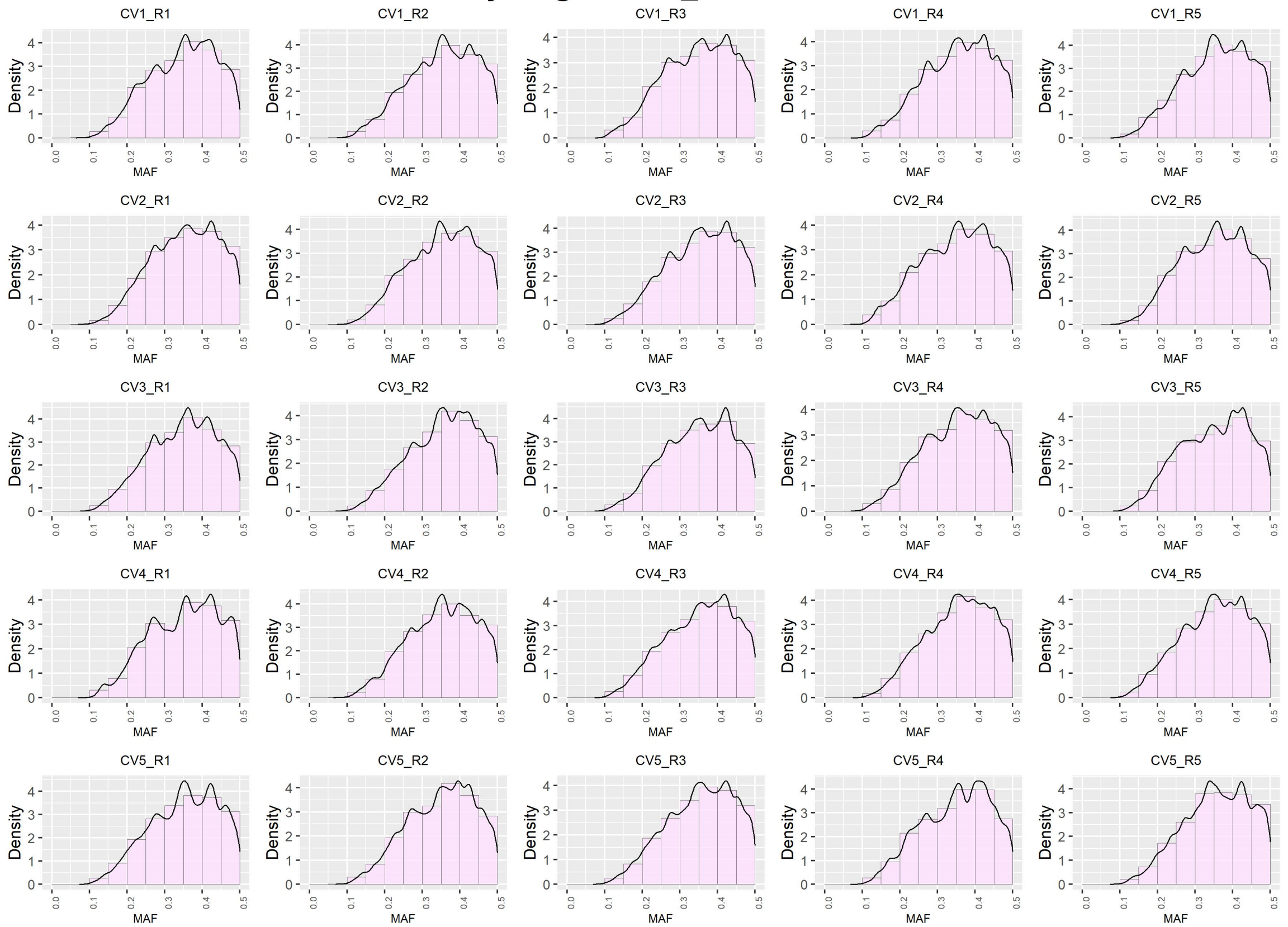

## (B) - Body length - 1K\_wssGBLUP

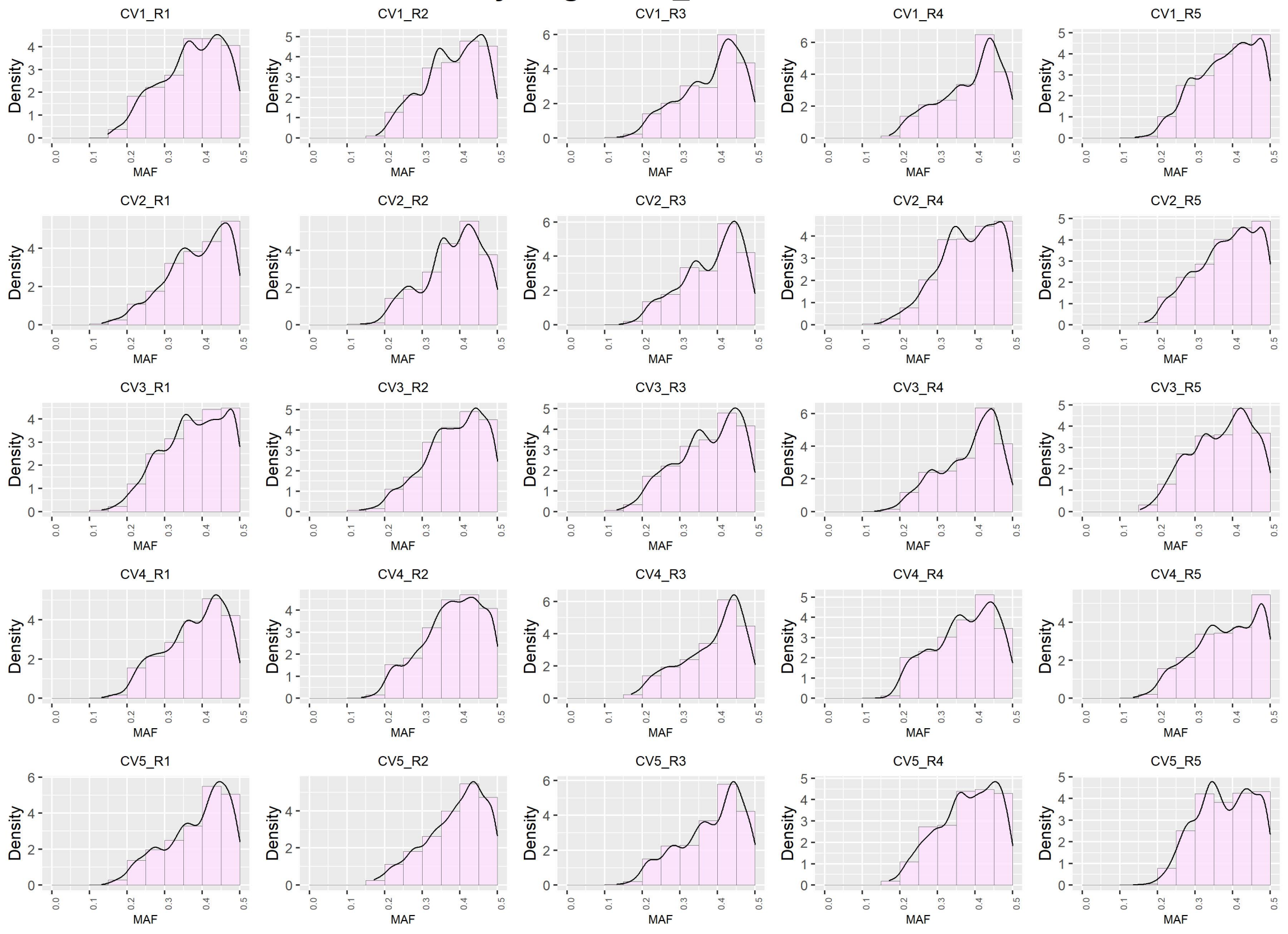

Supplement: Supplementary file 4 — Fig S4 [file EVA-15-537-s003.pdf]

**(A) - Body weight - 50K\_wssGBLUP**

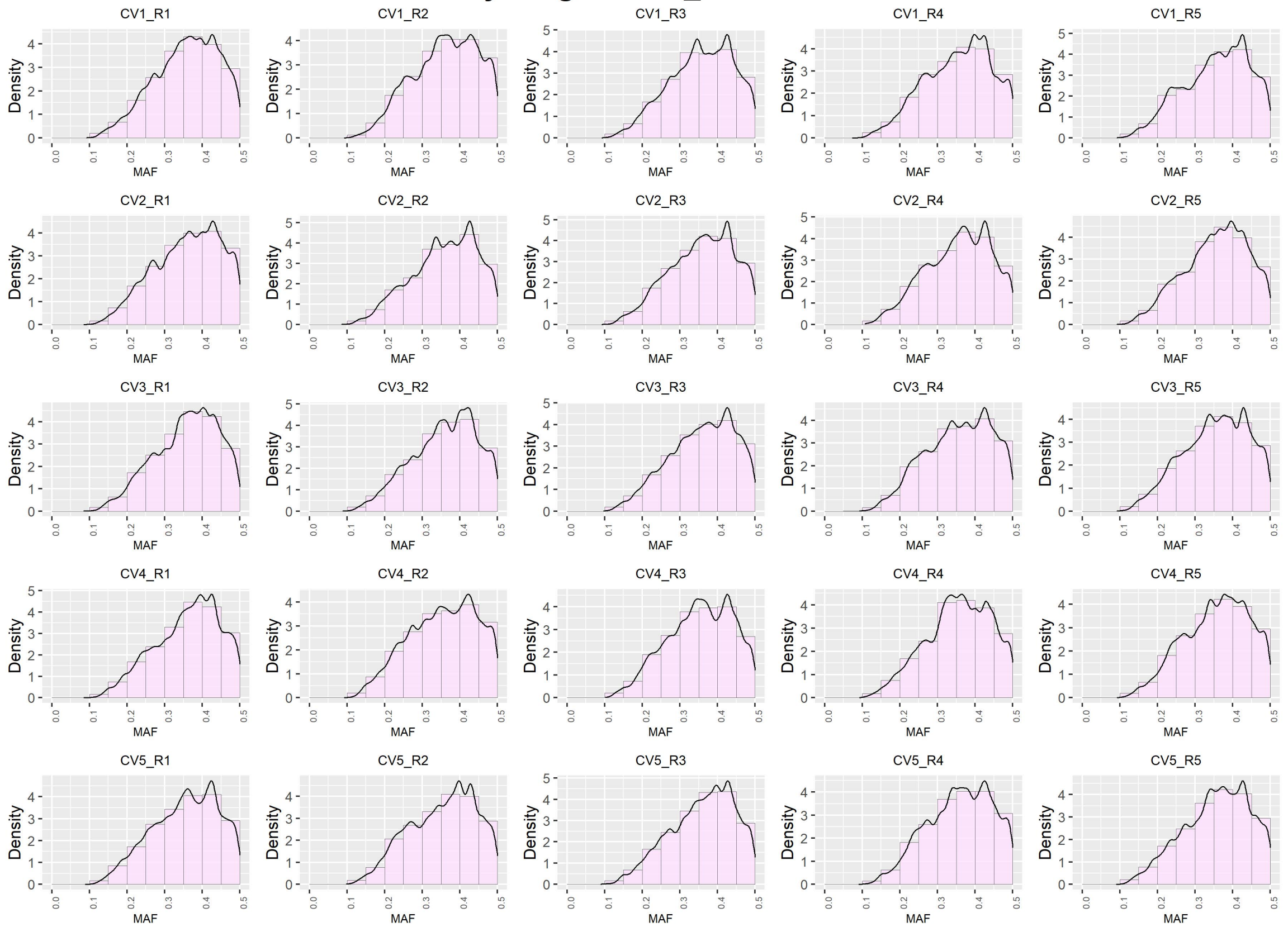

**(B) - Body weight - 1K\_wssGBLUP**

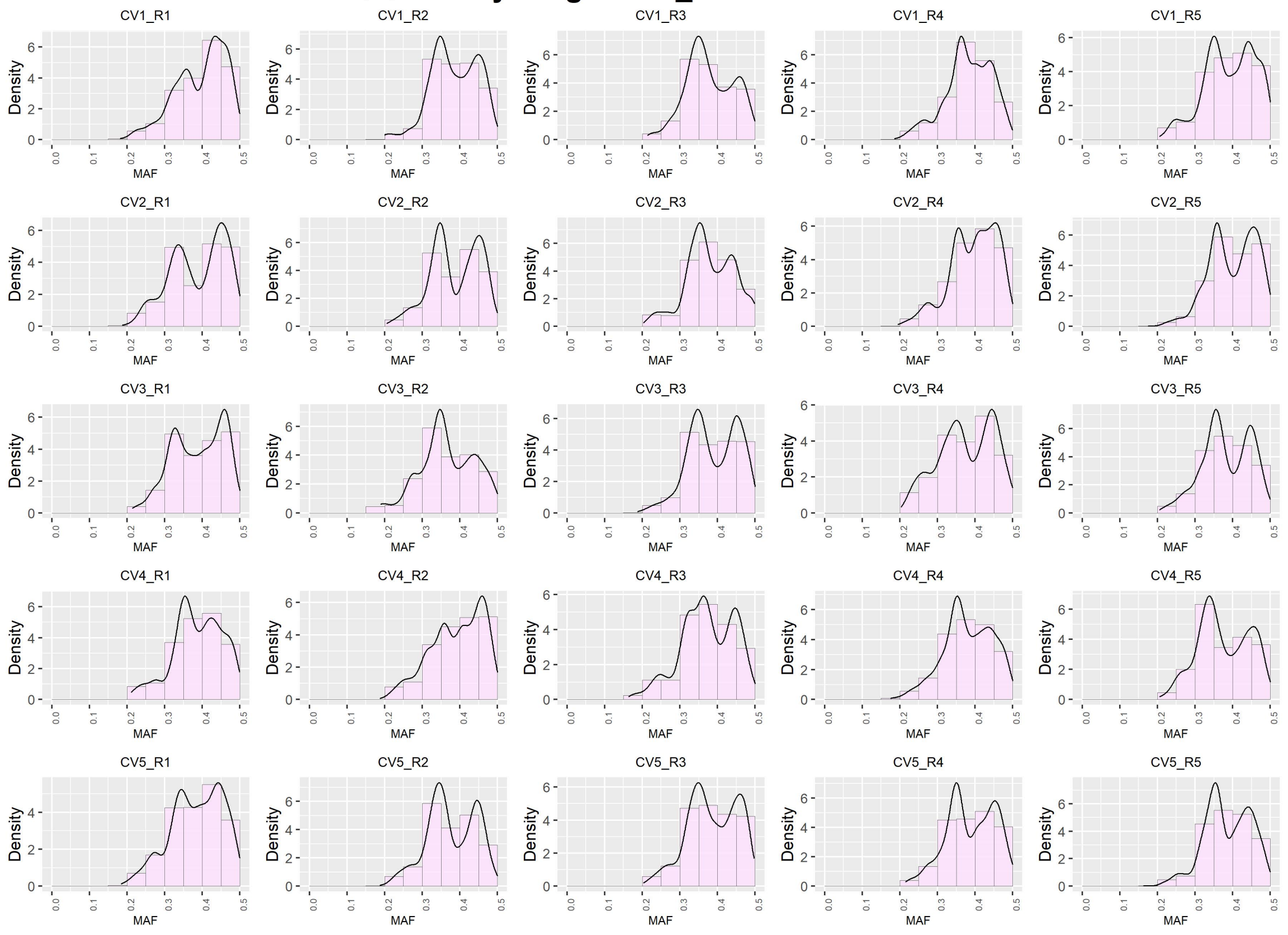

Supplement: Supplementary file 5 — Fig S5 [file EVA-15-537-s004.pdf]
